# Human microbiome privacy risks associated with summary statistics

**DOI:** 10.1371/journal.pone.0249528

**Published:** 2021-04-02

**Authors:** Jae-Chang Cho

**Affiliations:** Institute of Environmental Science and Department of Environmental Science, Hankuk University of Foreign Studies, Yong-In, Korea; Icahn School of Medicine at Mount Sinai, UNITED STATES

## Abstract

Recognizing that microbial community composition within the human microbiome is associated with the physiological state of the host has sparked a large number of human microbiome association studies (HMAS). With the increasing size of publicly available HMAS data, the privacy risk is also increasing because HMAS metadata could contain sensitive private information. I demonstrate that a simple test statistic based on the taxonomic profiles of an individual’s microbiome along with summary statistics of HMAS data can reveal the membership of the individual’s microbiome in an HMAS sample. In particular, species-level taxonomic data obtained from small-scale HMAS can be highly vulnerable to privacy risk. Minimal guidelines for HMAS data privacy are suggested, and an assessment of HMAS privacy risk using the simulation method proposed is recommended at the time of study design.

## Introduction

Humans have coevolved with an immense number of diverse microorganisms that inhabit our bodies, collectively referred to as the human microbiome [1]. Together with the development of metagenomics, recognizing that microbial community composition within the microbiome is associated with the physiological state of the host has sparked a large number of human microbiome association studies (HMAS), which are also referred to as human metagenome-wide association studies (MWAS) [2] in analogy to genome-wide association studies (GWAS) [3]. As in the field of GWAS [4–7], the privacy risk is increasing with the increasing size of publicly available HMAS data.

The privacy threats of HMAS data are based on the fact that individual microbiomes harbor personally identifiable information in the form of microbial community composition. Several prominent studies demonstrated that individual identity can be revealed using the human microbiome. Fierer et al. [8] showed that an individual who touched an object (e.g., computer keyboard) could be identified by matching the compositional profile of the microbiome on the surface of the object to that of the individual’s skin microbiome. While the authors’ approach can be properly applied to forensic analyses, similar microbiome-based approaches can also be used to reveal an individual’s location or intimate partner as shown by Lax et al. [9] and Kort et al. [10]. Besides, Franzosa et al. [11] presented the possibility of a different type of privacy threat that uses information stored in databases; the authors showed that metagenomic codes, as sets of differentiable features of any given microbiome, can be used to identify individuals in the Human Microbiome Project dataset.

Although the development of biological as well as computational/statistical tools for analyzing individual microbiomes helps us to better understand human microbiomes, such tools can be viewed as double-edged swords. The more a tool has resolving power, the more privacy risks confront people as described above. Unfortunately, we might not be able to prevent privacy threats by attackers who utilize microbiome-based forensic techniques because the attackers only need to seize microbiomes from victims. On the other hand, privacy threats by data breaches can be prevented when the HMAS community develops privacy-preserving methods for HMAS data analysis as shown by Wagner et al. [12] and by storing HMAS data in access-controlled databases such as the dbGaP [13], which is currently used as a secure database of human-related genotypic and phenotypic data. However, there is another type of privacy threat that can be caused by the publication of HMAS data. Although raw or detailed data are not presented, summary statistics (e.g., mean frequencies of prokaryotic taxa in study microbiomes) are frequently provided in tables and figures contained in HMAS-based papers. Concerns over privacy breaches due to publishing summary statistics was first raised by Homer et al. [14] with respect to GWAS privacy, and this type of privacy attack was later termed ’attribute disclosure attacks under the summary statistic scenario’ by Erlich and Narayanan [15]. To my knowledge, there have been no reports evaluating the privacy risk of HMAS summary statistics, which led me to perform a simple, foundational study in order to urge the HMAS community to be aware of privacy risks associated with HMAS dataset summary statistics.

In this paper, I demonstrate that the membership of an individual in the samples of an HMAS (e.g., case group or control group) can be revealed easily in the summary statistics of taxonomic compositions calculated from the microbiomes of the samples. Using a simple test statistic that was calculated from binary (presence/absence) taxonomic profiles, my simulation studies showed that publication of species-level taxonomic data obtained from small-scale HMAS can be highly vulnerable to privacy risk. This study asserts that the taxonomic profiles of the human microbiome should be treated as sensitive biometric information in that the HMAS metadata could contain the behavioral history of individuals in addition to medical conditions. I propose minimal guidelines for HMAS privacy and suggest that researchers use the simple simulation presented here to assess the privacy risk at the time of study design with the acknowledgment that a more advanced method could have greater resolving power for privacy breach.

## Methods

### Development of the test statistic

Suppose two samples *R* and *C*, each with size *n*_*R*_ and *n*_*C*_, were drawn independently from population P of human microbiomes. In HMAS, these samples may correspond to the sets of microbiomes of volunteers in the reference group (${\left\{{\vec{y}}_{i}^{R}\right\}}_{i=1}^{n_{R}}$) and case group (${\left\{{\vec{y}}_{i}^{C}\right\}}_{i=1}^{n_{C}}$), respectively; each microbiome is a vector of which the elements represent relative frequencies $y_{j}^{S}$ of *j* = 1,2,⋯,*t*, where *t* is the number of operational taxonomic units (OTUs) at different taxonomic levels from phylum to strain and *S*∈{*R*, *C*}. The summary statistics are vectors $\vec{r}$ and $\vec{c}$, of which the elements are the mean frequencies *r*_*j*_ and *c*_*j*_ of the OTUs in the pooled data obtained from *R* and *C*, respectively. Now consider the microbiome of an individual *q*, ${\vec{y}}^{q}$, of which the elements are simple binary measures of presence/absence of OUT *j* (i.e., $y_{j}^{q}\in \{0,1\}$), and suppose we want to determine whether ${\vec{y}}^{q}$ is a member of *R* or *C* using $\vec{r}$ and $\vec{c}$. First, calculate a distance *d* for OUT *j* using the absolute difference between $y_{j}^{q}$ and *c*_*j*_ and the absolute difference between $y_{j}^{q}$ and *r*_*j*_ as follows:

$$d_{j}=|y_{j}^{q}-r_{j}|-|y_{j}^{q}-c_{j}|$$

Assuming that OTUs are independent and invoking the central limit theorem for the large number of OTUs (*t*>50) examined in the HMAS, z-score of *d*_*j*_ across all OTUs will follow the standard normal distribution, *N*(0, 1).

$$Z=\frac{\bar{d}-\mu_{0}}{\sqrt{V(\bar{d})}}=\frac{\bar{d}-\mu_{0}}{\sqrt{V(d)/t}}\approx \frac{\bar{d}-\mu_{0}}{s/\sqrt{t}}∼N(0,1)$$

Since the *t* = max(*t*_*R*_, *t*_*C*_, *t*_*y*_) is large, the variance of *d* can be estimated reliably by the sample variance *s*^2^. The test statistic *Z* was inspired by Homer et al. [14] and Braun et al. [16]. The authors used a similar test statistic calculated from the single nucleotide polymorphism (SNP) genotyping data to identify an individual’s genotype in GWAS samples. I modified the original test statistic in order to use the binary taxonomic data in HMAS. Because an individual microbiome randomly drawn from population P should be equally distant from *R* and *C*, *μ*_0_ was presumably expected to be zero, i.e., *N*(0, 1) was a putative null distribution. Thus, under the null hypothesis $H_{0}:Z=0({\vec{y}}^{q}$ is a random draw from P), the alternative hypothesis $H_{R}:Z<0({\vec{y}}^{q}$ is a member of *R*) or $H_{C}:Z>0\;({\vec{y}}^{q}$ is a member of *C*) can be tested with an appropriate significance level *α*. For example, *Z*>1.65 rejects *H*_0_ in favor of *H*_*C*_ at *α* = 0.05 (one-tailed test).

### Distribution simulations

I examined the feasibility of the test statistic *Z* in identifying the presence of an individual’s microbiome in an HMAS sample with simulated datasets. Suppose that the OTUs correspond to species-level affiliations of microorganisms. Then, the presence of species *j* in P is a Bernoulli random variable with parameter *p*_*j*_. To model the probability distribution of *p*_*j*_ $\left(\vec{p}\right)$, I used four different *Beta*(*π*_1_, *π*_2_) distributions. For P with a small number of high-frequency species, *π*_1_ = 0.1 and *π*_2_ = 1.0 were assumed, and for P with a large number of high-frequency species, *π*_1_ = 1.0 and *π*_2_ = 0.1 were assumed. For P with a high number of high-frequency species as well as a high number of low-frequency species, *π*_1_ = 0.1 and *π*_2_ = 0.1 were assumed, which might be more realistic than the above two distributions in that the high-frequency species may correspond to constitutional or autochthonous prokaryotic populations and the low-frequency species may correspond to opportunistic or heterochthonous prokaryotic populations across individual microbiomes. In addition, a *Beta*(1, 1) (uniform) distribution was also used, which represents our ignorance of the distribution of *p*_*j*_ according to the principle of indifference.

Because the individual microbiome in *R* or *C* is a set of *t* random draws of *y*_*j*_~*Bernoulli*(*p*_*j*_), i.e., $\vec{y}∼Bernoulli(\vec{p})$, the summary statistics for samples *R* and *C* were simulated using $\vec{r}∼Binomial(n_{R},\vec{p})/n_{R}$ and $\vec{c}∼Binomial(n_{C},\vec{p})/n_{C}$, respectively. To construct density curves of true positives for samples *R* and *C*, random draws of ${\vec{y}}^{R+}$ and $y_{j}^{C+}$ from samples *R* and *C* were used to calculate the test statistics *Z*^*R*+^ and *Z*^*C*+^ $\left(n_{Z}^{R+}=n_{Z}^{C+}=100\right)$, respectively. Density curves were estimated using the Gaussian kernel density estimator. To construct density curves of the true null distribution, random draws of ${\vec{y}}^{P}$ from P were used to calculate test statistics *Z*^*P*^ $\left(n_{Z}^{P}=100\right)$. Python code for the simulation is available at https://colab.research.google.com/drive/1dOZi8OSo5qHmF7JPyGAP1I_BQdBVSSiC?usp=sharing.

## Results and discussion

### Overview of simulation results

**Fig 1:**
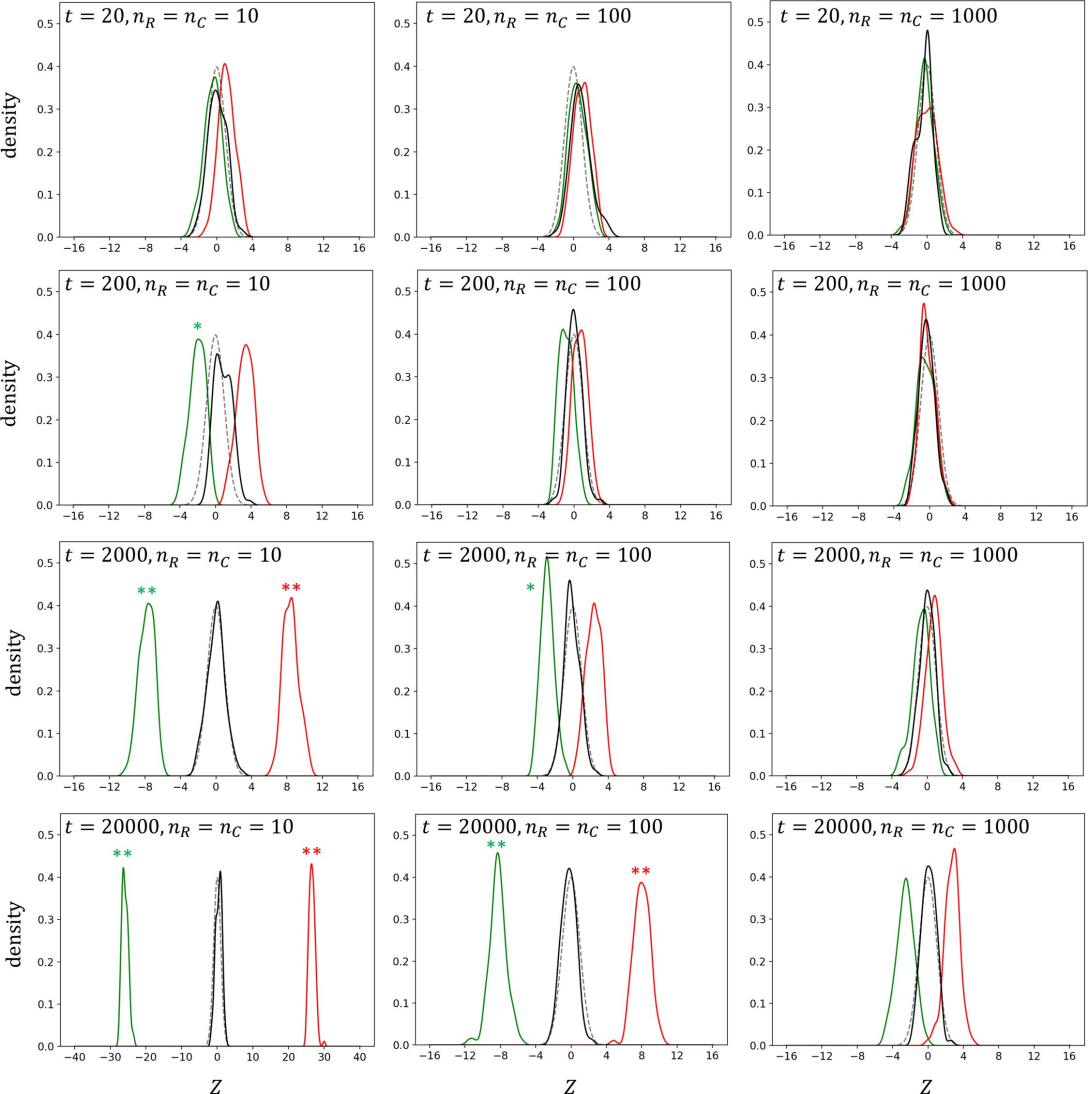
Distributions of the test statistic *Z* under the assumption that the population OTU frequencies follow a uniform (Beta(1, 1)) distribution. Density curves for true positives of samples *R* (*Z*^*R*+^) and *C* (*Z*^*C*+^) are denoted by green and red lines, respectively. Density curves of simulated null distribution and standard normal distribution are denoted by black and gray lines, respectively. Single and double asterisks represent type II error probabilities *β*<0.05 and *β*<0.01, respectively.

A simulation study was started with the number of OTUs *t* =2,000, which roughly reflects the number of species (including uncultivated candidate species) in the human gut microbiome [17], and with *n*_*R*_ = *n*_*C*_ = 10, which could correspond to small-scale HWAS, under the assumption that *p*_*j*_ follows uniform distribution (Fig 1). The null distribution estimated using *Z*^*P*^ was very close to *N*(0, 1) and crisply separated from the distributions of true positives (*Z*^*R*+^ and *Z*^*C*+^), indicating the feasibility of the microbiome-based identification. The distributions of true positives moved toward the null distribution with increasing *n* or with decreasing *t* but moved away from the null distribution with decreasing *n* or with increasing *t*. Similar distribution patterns were observed for all the underlying distributions of *p*_*j*_ assumed (S1–S3 Figs).

For numerical interpretation of the simulation results, I focused on the probability of type II error at *α* = 0.05 (*β*^*R*^ = *P*(*Z*^*R*+^>*z*_*α*_|*H*_*R*_) or (*β*^*C*^ = *P*(*Z*^*C*+^<*z*_*α*_|*H*_*C*_); the probability that the test statistic is not in the *H*_0_ rejection range, given that the alternative hypothesis is true). The power of the test (1−*β*) is important to a privacy attacker because it would be especially difficult for the attacker with a high *β* to determine whether the individual under investigation belongs to a particular HMAS sample due to the high false negative rate. *α* level critical values were obtained from percentiles of *Z*^*P*^ distribution because the true null distribution might diverge from *N*(0, 1). As expected in the density curves, *β* was far less than 0.01 for the experiments with a large *t* or small *n* (S1–S4 Tables).

**Fig 2:**
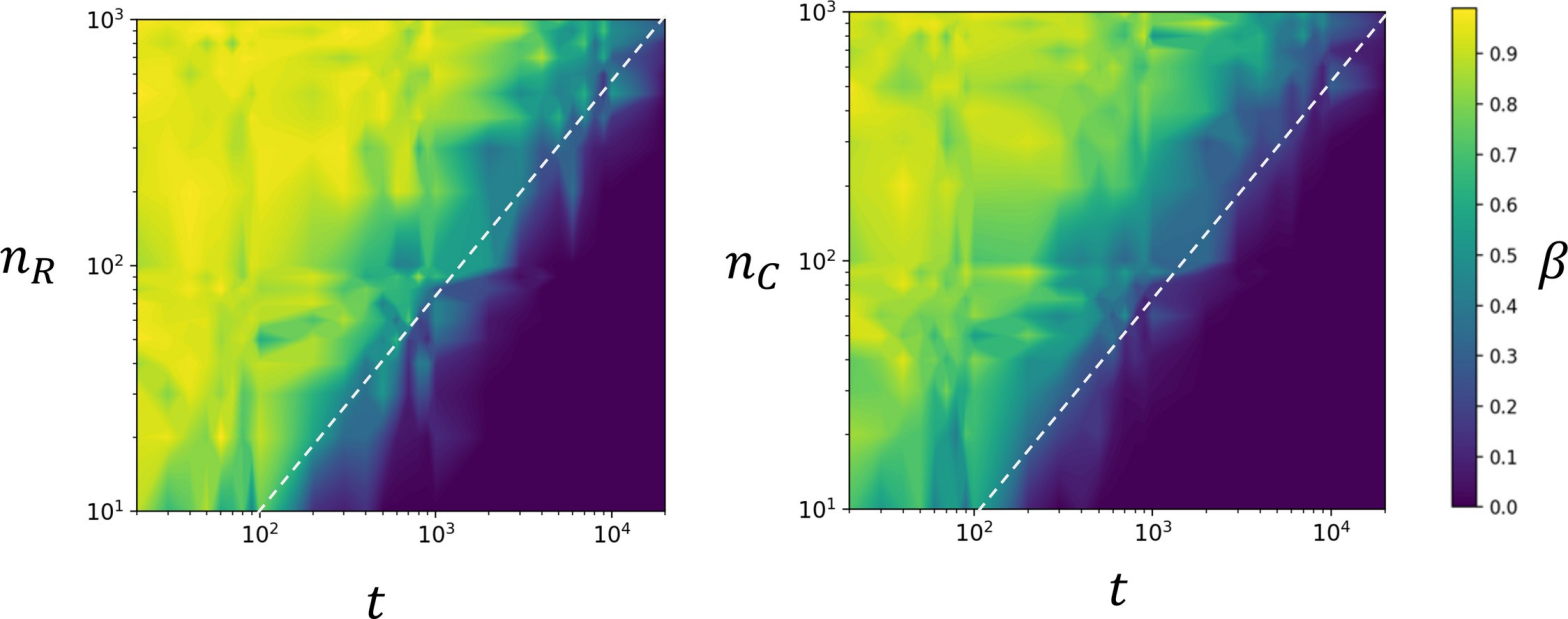
Contour plot representations of the type II error probabilities (*β*) for true positives of samples *R* and *C* under the assumption that the population OTU frequencies follow a uniform (*Beta*(1, 1)) distribution. Sample size and the number of OTUs are log-scaled. Dotted line denotes suggested minimal guidelines for HMAS privacy.

To formulate the guidelines for human microbiome privacy, the initial simulation study was expanded for virtual HMAS samples with *n*_*R*_ = *n*_*C*_ = 10, 20,…,100,…,1000 and with *t* = 20, 30,…,100,…,1000,…,20000. The method was in general slightly more powerful for *p*_*j*_ under the uniform distribution (Fig 2) than for *p*_*j*_ under other beta distributions (S4 and S5 Figs). In the resulting contour plots, the yellowish area represents higher *β* (i.e., lower test power); thus, the HMAS samples located in the dark blue area are considered to be vulnerable to privacy risk. The power of the test decreased notably with increasing HMAS sample size (*n*) or with a decreasing number of OTUs (*t*) from which the HMAS summary statistics are calculated, and it is possible to notice a borderline where *β* decreases considerably, which could help in assessing the privacy risk of the HMAS data.

### Effect of sample size

Samples of a large size approximate the population P because $\lim_{n_{R\to \infty }}r_{j}=p_{j}$ or $\lim_{n_{C\to \infty }}c_{j}=p_{j}$ (hence, $\lim_{{n_{R},n}_{C,\to \infty }}d_{j}=0$), and the distribution of *Z*^*R*+^ or *Z*^*C*+^ would overlap with the null distribution as shown in the selected density curves, indicating that a large sample size will make the classifying method ineffective. Contrarily, for the samples of a small size, $\bar{d}$ significantly deviates from *μ*_0_ even if the difference between *r*_*j*_ and *p*_*j*_ or between *c*_*j*_ and *p*_*j*_ is very small. Note that the sample size does not increase with the number of technical replicates used in HMAS data, since the sample points in technical replicates are not independent.

For an unequal sample size (e.g., *n*_*R*_ = 1,000 and *n*_*C*_ = 10), the samples with a larger size would approximate the null distribution, but the test power for identifying a microbiome in a sample with a smaller size would not be affected (S6 Fig). Considering that taxonomic profiles of individual microbiomes in the case sample could be more homogeneous than those in the control sample, the effective size of the case sample might be smaller than the census size of the case sample. This would result in much higher statistical power for testing whether an individual is a member of the case sample, which is a primary interest of the attackers.

### Effect of the number of OTUs

With the sample size fixed, an increased number of OTUs would increase the test power. This situation can happen when the HMAS data contains taxonomic profiles with a resolution finer than species level. Under the arbitrary speculation that each species is comprised of ca. 10 strains (*t* = 20,000), the method was very powerful (*β*≪0.01) even for a moderate sample size. This is because the denominator of the test statistic shrinks by increasing *t* compared to the sample mean $\bar{d}$, which results in a large *Z*^*R*+^ or *Z*^*C*+^. In the same vein, a reduced number (e.g., *t* = 20) of OTUs (roughly corresponding to near phylum-level) would decrease the power of the test.

### Effect of correlation among OTUs

The occurrence of many OTUs in the human microbiome could be correlated, since the microbiome itself is an ecological community [18]. If the correlation among OTUs is significant, the violation of the assumption that OTUs are independent could change the test power. I analytically evaluated the effect of the OUT correlation on test power. If OTUs are not independent, the variance of $\bar{d}$ includes covariance (*Cov*) terms as follows:

$$V(\bar{d})=\frac{1}{t}V(d)+\frac{2}{t^{2}}\sum_{j}^{t}\sum_{j^{\prime }}^{t}Cov(d_{j},d_{j^{\prime }}),$$

where *j*<*j*′. By letting $\tilde{\rho }$ be the average correlation among *d*_*j*_ such that $V(\bar{d})\geq 0$, the above equation can be written as follows:

$$V(\bar{d})=\frac{V(d)}{t/(1+\tilde{\rho }t-\tilde{\rho })},$$

where $1+\tilde{\rho }t-\tilde{\rho }\geq 0$. Thus, $V(\bar{d})$ increases if $\tilde{\rho }$ is positive ($0<\tilde{\rho }\leq 1⟹V(d)/t<V(\bar{d})\leq V(d))$ or decreases if $\tilde{\rho }$ is negative $(-1/(t-1)\leq \tilde{\rho }<0⟹0\leq V(\bar{d})\leq V(d)/t).$ One can imagine that the changes in $V(\bar{d})$ result in changes in the distribution of test statistic *Z*. But the effect of $\tilde{\rho }$ on test power is not easy to see with changes in $V(\bar{d})$. Rather, we can view the denominator term of $V(\bar{d})$ as an effective number of independent OUTs (*t*_*e*_) as follows:

$$t_{e}=\frac{t}{1+\tilde{\rho }t-\tilde{\rho }}$$

Note that *t*_*e*_ = *t* if $\tilde{\rho }=0$, 1≤*t*_*e*_<*t* if $0<\tilde{\rho }\leq 1$, and *t*<*t*_*e*_ if $-1/(t-1)\leq \tilde{\rho }<0$ (*t_e_*→∞ as $\tilde{\rho }\to -1/(t-1)$). For example, if three of 10 OTUs show a strong positive correlation, two of these OTUs cannot contribute to the number of OTUs as equally as other independent OTUs. Thus, the effect of a positive $\tilde{\rho }$ could be similar to the effect of a decreased number of OTUs, resulting in decreased test power as in the case of linkage disequilibrium among SNPs [16]. However, $\tilde{\rho }$ can also be negative if negative interactions among OTUs are more prevalent than positive interactions among OTUs, which could subsequently increase test power. Even very small negative correlations (e.g., $\tilde{\rho }=-0.001$) among a moderate number of OTUs (*t* = 1000) would increase *t*_*e*_ to 10^6^, while very small positive correlations (e.g., $\tilde{\rho }=+0.001$) decrease *t*_*e*_ to 500. The negative correlation overwhelmingly affects *t*_*e*_ because *t*_*e*_ is a reciprocal function of $\tilde{\rho }$ when *t* is fixed (S7 Fig). Nonetheless, because $\tilde{\rho }$ cannot be measured from summary statistics unless provided or assumed, the effect of $\tilde{\rho }$ should be investigated more comprehensively in the future.

### Additional considerations

I used the binary vector as a query microbiome because it was considered that the binary data is less prone to experimental variations. If a frequency vector is used, the $\bar{d}$ could increase because small differences between $\left|y_{j}^{q}-r_{j}\right|$ and |$y_{j}^{q}-c_{j}$| can accumulate across a large number of OTUs. However, it would not always result in a larger *Z*^*R*+^ or *Z*^*C*+^ because the increased sample variance could counteract the increase in $\bar{d}$. Thus, the use of a frequency vector might not guarantee more test power, rather it could make $\bar{d}$ more prone to errors in OTU frequencies.

It was assumed that *R* and *C* were samples drawn independently from the population of human microbiomes (P). Violation of this assumption could shift the location of the null distribution (*μ*_0_). Suppose that populations underlying samples *R* and *C* (denoted by R and C, respectively) are all different from the population underlying ${\vec{y}}^{q}(P)$. If the systemic difference between R and C is significant, the difference between summary statistics can deviate from zero, i.e., *E*(Δ) = *E*(*r*−*c*)≠0. Let $d_{j}^{*}$ be the distance metric that includes Δ term as follows:

dj*=|yjq−Δj−cj|−|yjq−cj|={Δjforyjq=0−Δjforyjq=1

Then, according Braun et al. [16] and using $P(y_{j}^{q}=0|p_{j})=1-p_{j}$ and $P(y_{j}^{q}=1|p_{j})=p_{j}$, the expected value of the distance metric under the null hypothesis is as follows:

$$\mu_{0}^{*}=E(\Delta -2\Delta p)=E(\Delta )-2E(\Delta )E(p)$$

Because −1≤*E*(Δ)≤1 and $0<E(p)\leq 1,\mu_{0}^{*}$ ranges from -1 to 1 which is quite small compared to the distance between the distributions of *Z*^*P*^ and *Z*^*R*+^ or between the distributions of *Z*^*P*^ and *Z*^*C*+^ in the cases where the test power is very high (e.g., *t*≥2000 and *n* = 10 or *t*≥20000 and *n* = 100) (Fig 1). Moreover, as *E*(*p*) deviates from its two extreme values (0 or 1) which are highly unrealistic in microbiome composition, the 2*E*(Δ)*E*(*p*) term counteracts the deviation of $\mu_{0}^{*}$ from zero (i.e., $\mu_{0}^{*}\to 0$ as *E*(*p*)→0.5). Thus, I considered that the violation of the assumption that underlying populations are all different would not alter the main results of this study. In fact, if *R* (reference/control group) and *C* (case/treatment group) are assumed to be samples drawn from P and C, respectively, the test statistic would become very similar to that used for a two-tailed z-test (or t-test) which can be used to test the null hypothesis that $y_{j}^{q}$ is not a member of *C*.

Although it might be virtually impossible for the HMAS community to develop an almighty shield that protects data against all possible privacy breaching methods, we do not need to overreact to HMAS privacy concerns since the taxonomic profile of the individual’s microbiome could not be a permanent identifier, while a subset of an individual’s microbiome may endure for most of the lifetime. Nonetheless, the HMAS community should have guidelines for reducing the privacy risk, which could be kept minimal in order not to obstruct our understanding of the human microbiome.

### Concluding remarks

This study showed the possibilities of privacy breaches using the summary statistics drawn from HMAS data. The key finding was that the publication of species composition data obtained from small-scale HMAS (e.g., *t*≈1,000 and *n*_*R*_ = *n*_*C*_ = 10) easily exposes the privacy of the victims. The HMAS samples with moderate size (*n*_*R*_ = *n*_*C*_≈100) and *t*≈1000 were on the vicinity of the borderline. I suggest these figures as a basis for HMAS data release policy while acknowledging a sophisticated test statistic that employs better distance metric along with the violation of underlying assumptions could improve the power of the privacy breaching methods. I propose minimal guidelines for HMAS data release as follows: i) increase the sample size as much as manageable, ii) do not publish species composition data even in the form of summary statistics if the sample size is less than 10, iii) avoid publishing subspecies- or strain-level data unless the sample size is far larger than 100. I also suggest that HMAS researchers evaluate the privacy risk at the time of study design using the simulation method presented in this study. Because the test statistic used was relatively robust against the variations in the models for the distribution of *p*_*j*_, HMAS researchers can simply use uniform distribution and their intended *t*, *n*_*R*_, and *n*_*C*_ to estimate the ‘minimal’ power of the privacy-breaching method.

This study focused only on attribute disclosure attacks under the summary statistic scenario [15]. However, HMAS data privacy concerns are not limited to summary statistics; privacy breaches can occur in various manners at any stage of HMAS data management as described by Wagner et al. [12]. Thus, it is timely that the HMAS community begins comprehensive discussions on HMAS data privacy risks and developing privacy-preserving algorithms for data storage and release.

## Supporting information

S1 Fig Distributions of test statistic *Z* under the assumption that population OTU frequencies follow a *Beta*(0.1, 1) distribution. Density curves for true positives of samples *R* (*Z*^*R*+^) and *C* (*Z*^*C*+^) are denoted by green and red lines, respectively. Density curves of simulated null distribution and standard normal distribution are denoted by black and gray lines, respectively. Single and double asterisks represent type II error probabilities *β*<0.05 and *β*<0.01, respectively.
(PDF)

S2 Fig Distributions of the test statistic *Z* under the assumption that population OTU frequencies follow a *Beta*(1, 0.1) distribution. Density curves for true positives of samples *R* (*Z*^*R*+^) and *C* (*Z*^*C*+^) are denoted by green and red lines, respectively. Density curves of simulated null distribution and standard normal distribution are denoted by black and gray lines, respectively. Single and double asterisks represent type II error probabilities *β*<0.05 and *β*<0.01, respectively.
(PDF)

S3 Fig Distributions of the test statistic *Z* under the assumption that population OTU frequencies follow a *Beta*(0.1, 0.1) distribution. Density curves for true positives of samples *R* (*Z*^*R*+^) and *C* (*Z*^*C*+^ are denoted by green and red lines, respectively. Density curves of simulated null distribution and standard normal distribution are denoted by black and gray lines, respectively. Single and double asterisks represent type II error probabilities *β*<0.05 and *β*<0.01, respectively.
(PDF)

S4 Fig Contour plot representations of the type II error probabilities (*β*) for true positives of sample *R* under the assumptions that population OTU frequencies follow *Beta*(1, 1), *Beta*(0.1, 1), *Beta*(1, 0.1) and *Beta*(0.1, 0.1) distributions. Sample size and the number of OTUs are log-scaled. Dotted line denotes suggested minimal guidelines for HMAS privacy.
(PDF)

S5 Fig Contour plot representations of the type II error probabilities (*β*) for true positives of sample *C* under the assumptions that population OTU frequencies follow *Beta*(1, 1), *Beta*(0.1, 1), *Beta*(1, 0.1) and *Beta*(0.1, 0.1) distributions. Sample size and the number of OTUs are log-scaled. Dotted line denotes suggested minimal guidelines for HMAS privacy.
(PDF)

S6 Fig Distributions of the test statistic *Z* with unequal sample sizes under the assumption that population OTU frequencies follow a *Beta*(1, 1) distribution. Density curves for true positives of samples *R* (*Z*^*R*+^) and *C* (*Z*^*C*+^) are denoted by green and red lines, respectively. Density curves of simulated null distribution and standard normal distribution are denoted by black and gray lines, respectively. Single and double asterisks represent type II error probabilities *β*<0.05 and *β*<0.01, respectively.
(PDF)

S7 Fig Relationship between the effective number of OTUs and the average correlation among OTUs. (PDF)

S1 Table Summary statistics of simulation results obtained under the assumption that the population OTU frequencies follow a *Beta*(1, 1) distribution. Type II error probabilities less than 0.05 are in bold.
(PDF)

S2 Table Summary statistics of simulation results obtained under the assumption that the population OTU frequencies follow a *Beta*(0.1, 1) distribution. Type II error probabilities less than 0.05 are in bold.
(PDF)

S3 Table Summary statistics of simulation results obtained under the assumption that the population OTU frequencies follow a *Beta*(1, 0.1) distribution. Type II error probabilities less than 0.05 are in bold.
(PDF)

S4 Table Summary statistics of simulation results obtained under the assumption that the population OTU frequencies follow a *Beta*(1, 1) distribution. Type II error probabilities less than 0.05 are in bold.
(PDF)
