# Supplementary material for: Human microbiome privacy risks associated with summary statistics
Source: PLoS One. 2021 Apr 2;16(4):e0249528. doi: 10.1371/journal.pone.0249528 (PMC8018636; doi:10.1371/journal.pone.0249528)
Supplement: S1 Fig — Density curves for true positives of samples R (ZR+) and C (ZC+) are denoted by green and red lines, respectively. Density curves of simulated null distribution and standard normal distribution are denoted by black and gray lines, respectively. Single and double asterisks represent type II error probabilities β<0.05 and β<0.01, respectively. (PDF) [file pone.0249528.s001.pdf]

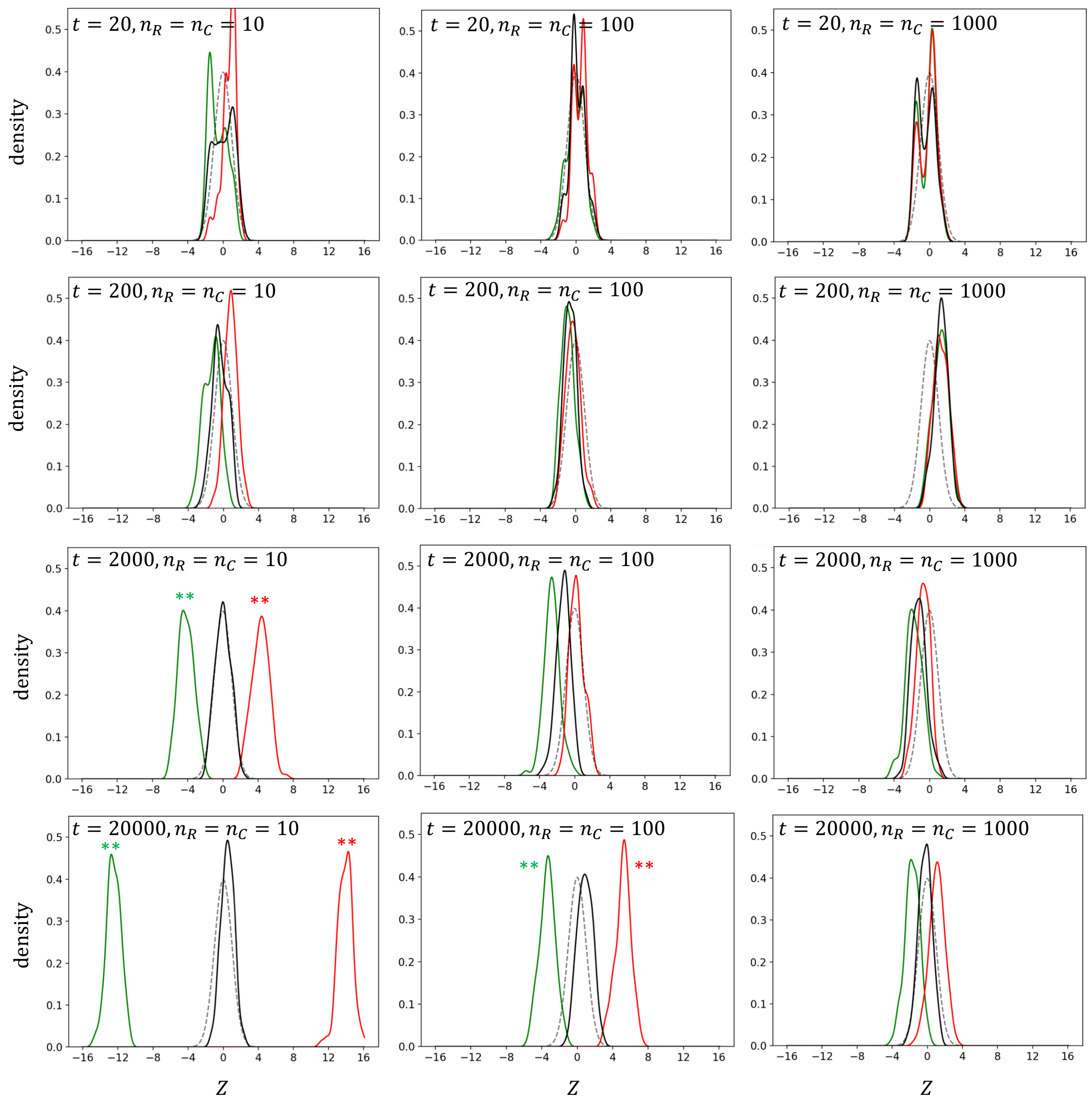

S1 Fig. Distributions of test statistic Z under the assumption that population OTU frequencies follow a $\boldsymbol{Beta}(\mathbf{0.1}, \mathbf{1})$ distribution. Density curves for true positives of samples R (Z^{R+}) and C (Z^{C+}) are denoted by green and red lines, respectively. Density curves of simulated null distribution and standard normal distribution are denoted by black and gray lines, respectively. Single and double asterisks represent type II error probabilities $\beta < 0.05$ and $\beta < 0.01$, respectively.
