# Supplementary material for: Human microbiome privacy risks associated with summary statistics
Source: PLoS One. 2021 Apr 2;16(4):e0249528. doi: 10.1371/journal.pone.0249528 (PMC8018636; doi:10.1371/journal.pone.0249528)
Supplement: S4 Fig — Sample size and the number of OTUs are log-scaled. Dotted line denotes suggested minimal guidelines for HMAS privacy. (PDF) [file pone.0249528.s004.pdf]

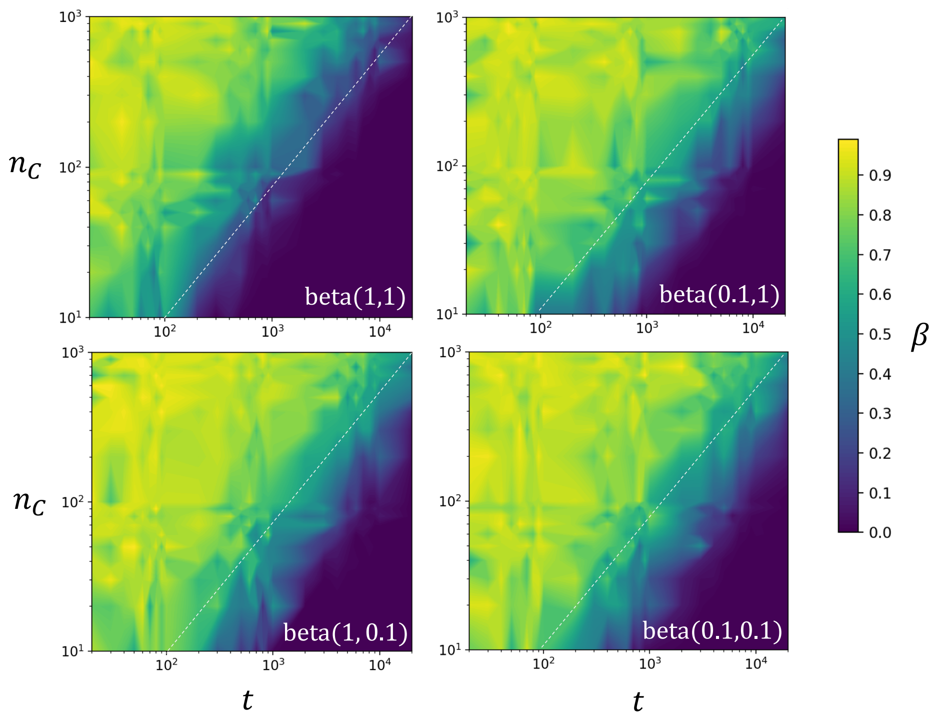

S4 Fig. Contour plot representations of the type II error probabilities ($\boldsymbol{\beta}$) for true positives of sample $\boldsymbol{R}$ under the assumptions that population OTU frequencies follow $\boldsymbol{Beta(1,1)}$, $\boldsymbol{Beta(0.1,1)}$, $\boldsymbol{Beta(1,0.1)}$, and $\boldsymbol{Beta(0.1,0.1)}$ distributions. Sample size and the number of OTUs are log-scaled. Dotted line denotes suggested minimal guidelines for HMAS privacy.
