# Supplementary material for: Human microbiome privacy risks associated with summary statistics
Source: PLoS One. 2021 Apr 2;16(4):e0249528. doi: 10.1371/journal.pone.0249528 (PMC8018636; doi:10.1371/journal.pone.0249528)
Supplement: S5 Fig — Sample size and the number of OTUs are log-scaled. Dotted line denotes suggested minimal guidelines for HMAS privacy. (PDF) [file pone.0249528.s005.pdf]

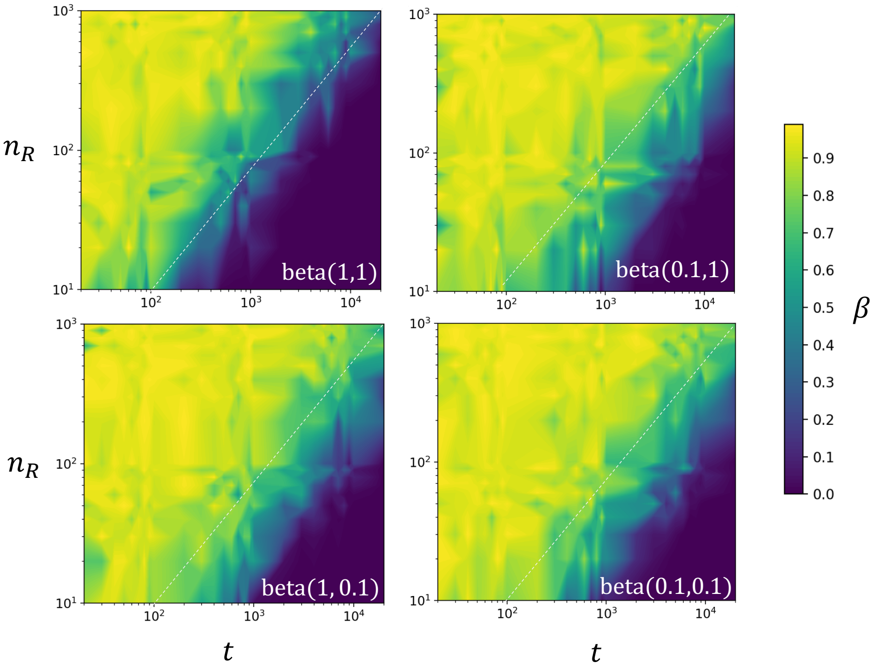

S5 Fig. Contour plot representations of the type II error probabilities ($\boldsymbol{\beta}$) for true positives of sample $\boldsymbol{C}$ under the assumptions that population OTU frequencies follow $\boldsymbol{Beta}(\mathbf{1},\mathbf{1})$, $\boldsymbol{Beta}(\mathbf{0.1},\mathbf{1})$, $\boldsymbol{Beta}(\mathbf{1},\mathbf{0.1})$, and $\boldsymbol{Beta}(\mathbf{0.1},\mathbf{0.1})$ distributions. Sample size and the number of OTUs are log-scaled. Dotted line denotes suggested minimal guidelines for HMAS privacy.
