# Supplementary material for: Human microbiome privacy risks associated with summary statistics
Source: PLoS One. 2021 Apr 2;16(4):e0249528. doi: 10.1371/journal.pone.0249528 (PMC8018636; doi:10.1371/journal.pone.0249528)
Supplement: S7 Fig — (PDF) [file pone.0249528.s007.pdf]

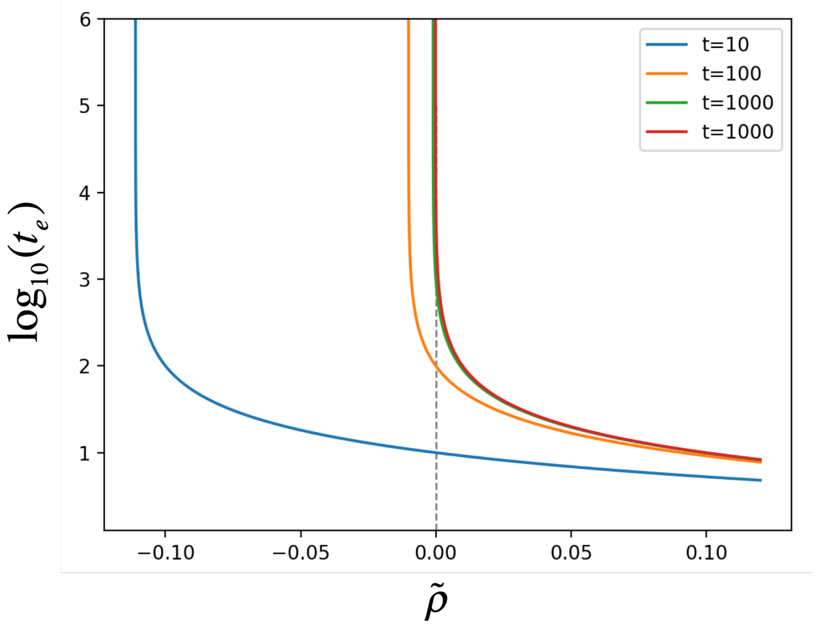

S7 Fig. Relationship between the effective number of OTUs and the average correlation among OTUs.
