# Supplementary material for: Human microbiome privacy risks associated with summary statistics
Source: PLoS One. 2021 Apr 2;16(4):e0249528. doi: 10.1371/journal.pone.0249528 (PMC8018636; doi:10.1371/journal.pone.0249528)
Supplement: S1 Table — Type II error probabilities less than 0.05 are in bold. (PDF) [file pone.0249528.s008.pdf]

S1 Table. Summary statistics of simulation results obtained under the assumption that the population OTU frequencies follow a $Beta(1,1)$ distribution. Type II error probabilities less than 0.05 are in bold.

			$n_R = n_C = 10$			$n_R = n_C = 100$			$n_R = n_C = 1000$		
			Z^P	Z^{R+}	Z^{C+}	Z^P	Z^{R+}	Z^{C+}	Z^P	Z^{R+}	Z^{C+}
t = 20	Mean		0.08	-0.42	1.11	0.82	0.49	1.03	-0.35	-0.26	0.00
	Standard deviation		1.03	0.98	0.91	1.13	0.98	0.93	0.90	0.96	1.11
	Percentile	5%	-1.64	-2.06	-0.31	-0.79	-0.87	-0.32	-1.93	-1.61	-1.70
		95%	1.64	1.28	2.47	3.12	1.96	2.49	1.08	1.39	1.75
	β	*N* (0, 1)		0.8746	0.7091		0.9824	0.7188		0.9168	0.9127
		Z^P		0.9555	0.8795		0.8888	0.9885		0.9498	0.8097
t = 200	Mean		0.86	-2.01	3.32	0.06	-0.88	0.69	-0.33	-0.46	-0.21
	Standard deviation		0.96	0.88	0.93	0.84	0.80	0.82	0.85	0.99	0.85
	Percentile	5%	-0.47	-3.57	1.62	-1.14	-2.06	-0.63	-1.77	-2.11	-1.52
		95%	2.34	-0.84	4.69	1.36	0.53	2.08	1.04	1.17	1.17
	β	*N* (0, 1)		0.3801	0.0593		0.7921	0.8539		0.8686	0.9708
		Z^P		**0.0384**	0.1714		0.5895	0.7658		0.8913	0.9044
t = 2000	Mean		0.03	-7.82	8.44	-0.09	-2.86	2.36	-0.01	-0.69	0.76
	Standard deviation		0.99	0.84	0.87	0.87	0.75	0.84	0.86	0.97	0.94
	Percentile	5%	-1.78	-9.20	7.29	-1.39	-4.06	0.97	-1.47	-2.71	-0.81
		95%	1.67	-6.65	9.99	1.22	-1.64	3.66	1.20	0.89	2.22
	β	*N* (0, 1)		**< 0.0001**	**< 0.0001**		0.0745	0.2321		0.8298	0.8265
		Z^P		**< 0.0001**	**< 0.0001**		**0.0429**	0.1152		0.7867	0.6780
t = 20000	Mean		0.44	-25.87	26.60	-0.25	-8.27	8.01	0.11	-2.55	2.72
	Standard deviation		0.88	0.86	0.84	0.85	0.95	0.92	0.81	0.97	0.85
	Percentile	5%	-1.00	-27.17	25.29	-1.57	-9.70	6.55	-1.15	-4.19	1.47
		95%	1.70	-24.51	27.89	0.93	-6.57	9.47	1.36	-1.05	4.05
	β	*N* (0, 1)		**< 0.0001**	**< 0.0001**		**< 0.0001**	**< 0.0001**		0.1954	0.1091
		Z^P		**< 0.0001**	**< 0.0001**		**< 0.0001**	**< 0.0001**		0.0891	0.0672
