# Supplementary material for: Human microbiome privacy risks associated with summary statistics
Source: PLoS One. 2021 Apr 2;16(4):e0249528. doi: 10.1371/journal.pone.0249528 (PMC8018636; doi:10.1371/journal.pone.0249528)
Supplement: S2 Table — Type II error probabilities less than 0.05 are in bold. (PDF) [file pone.0249528.s009.pdf]

S2 Table. Summary statistics of simulation results obtained under the assumption that the population OTU frequencies follow a $\boldsymbol{Beta(0.1, 1)}$ distribution. Type II error probabilities less than 0.05 are in bold.

			$n_R = n_C = 10$			$n_R = n_C = 100$			$n_R = n_C = 1000$		
			Z^P	Z^{R+}	Z^{C+}	Z^P	Z^{R+}	Z^{C+}	Z^P	Z^{R+}	Z^{C+}
t = 20	Mean		0.08	-0.56	0.69	0.18	-0.07	0.49	-0.42	-0.27	-0.18
	Standard deviation		1.12	1.00	0.75	0.85	0.95	0.82	0.96	0.96	0.96
	Percentile	5%	-1.49	-1.51	-0.59	-1.43	-1.43	-0.50	-1.59	-1.59	-1.59
		95%	1.83	1.17	1.83	1.88	1.03	1.88	1.14	1.14	1.14
	β	*N* (0, 1)		0.8275	0.9307		0.9282	0.8964		0.8779	0.9782
		Z^P		0.7579	0.9638		0.8896	0.9326		0.8581	0.9193
t = 200	Mean		-0.38	-1.33	0.84	-0.63	-0.89	-0.28	1.31	1.20	1.30
	Standard deviation		0.86	0.91	0.73	0.72	0.76	0.82	0.78	0.85	0.84
	Percentile	5%	-1.80	-2.78	-0.29	-1.69	-2.06	-1.58	-0.13	-0.20	-0.07
		95%	1.04	0.10	2.00	0.40	0.44	1.12	2.42	2.50	2.63
	β	*N* (0, 1)		0.6249	0.8488		0.8154	0.9745		> 0.9999	0.6435
		Z^P		0.6696	0.5989		0.8279	0.7882		0.9111	0.8812
t = 2000	Mean		0.03	-4.18	4.27	-1.30	-2.60	0.23	-1.21	-1.74	-0.73
	Standard deviation		0.86	0.87	0.95	0.78	0.87	0.81	0.85	0.94	0.76
	Percentile	5%	-1.37	-5.67	2.65	-2.56	-3.96	-0.92	-2.35	-3.27	-2.04
		95%	1.47	-2.70	5.68	-0.06	-1.10	1.65	0.32	-0.23	0.31
	β	*N* (0, 1)		**0.0015**	**0.0017**		0.1379	0.9300		0.4573	0.9998
		Z^P		**0.0002**	**0.0005**		0.4774	0.3887		0.7335	0.9077
t = 20000	Mean		0.49	-12.49	13.90	0.88	-3.36	5.18	-0.24	-1.71	1.10
	Standard deviation		0.74	0.80	0.82	0.83	0.87	0.86	0.75	0.82	0.88
	Percentile	5%	-0.56	-13.75	12.80	-0.42	-4.82	3.62	-1.48	-3.26	-0.43
		95%	1.56	-11.21	15.18	2.17	-1.88	6.60	0.98	-0.40	2.46
	β	*N* (0, 1)		**< 0.0001**	**< 0.0001**		**0.0360**	**< 0.0001**		0.4829	0.7197
		Z^P		**< 0.0001**	**< 0.0001**		**0.0001**	**0.0001**		0.4080	0.4402
