# Supplementary material for: Human microbiome privacy risks associated with summary statistics
Source: PLoS One. 2021 Apr 2;16(4):e0249528. doi: 10.1371/journal.pone.0249528 (PMC8018636; doi:10.1371/journal.pone.0249528)
Supplement: S3 Table — Type II error probabilities less than 0.05 are in bold. (PDF) [file pone.0249528.s010.pdf]

S3 Table. Summary statistics of simulation results obtained under the assumption that the population OTU frequencies follow a $\boldsymbol{Beta(1, 0.1)}$ distribution. Type II error probabilities less than 0.05 are in bold.

			$n_R = n_C = 10$			$n_R = n_C = 100$			$n_R = n_C = 1000$		
			Z^P	Z^{R+}	Z^{C+}	Z^P	Z^{R+}	Z^{C+}	Z^P	Z^{R+}	Z^{C+}
t = 20	Mean		0.29	-0.05	0.83	-0.21	-0.30	0.17	-0.59	-0.50	-0.27
	Standard deviation		0.93	0.84	0.79	0.89	0.83	1.09	0.72	0.82	0.81
	Percentile	5%	-1.03	-1.59	-0.50	-1.54	-1.66	-1.55	-1.88	-1.34	-1.43
		95%	2.24	1.59	1.59	1.10	0.89	2.03	0.63	1.13	1.13
	β	*N* (0, 1)		0.9612	0.8862		0.9208	0.8936		0.9429	0.9795
		Z^P		0.8274	0.9784		0.9010	0.7847		0.9781	0.8509
t = 200	Mean		-0.13	-1.47	0.87	-0.59	-1.03	-0.33	0.39	0.22	0.29
	Standard deviation		0.89	0.82	0.85	0.74	0.86	0.78	0.79	0.83	0.76
	Percentile	5%	-1.50	-2.68	-0.49	-1.77	-2.35	-1.67	-0.94	-1.23	-0.87
		95%	1.33	-0.31	2.16	0.38	0.55	0.91	1.56	1.61	1.59
	β	*N* (0, 1)		0.5929	0.5352		0.7347	0.9935		0.9744	0.9419
		Z^P		0.7908	0.6793		0.7766	0.7965		0.8988	0.9267
t = 2000	Mean		0.28	-3.59	4.45	-0.72	-1.97	0.76	0.14	-0.36	0.49
	Standard deviation		0.75	0.78	0.73	0.69	0.87	0.79	0.83	0.84	0.80
	Percentile	5%	-0.89	-4.84	3.40	-1.74	-3.31	-0.61	-1.17	-1.82	-0.71
		95%	1.46	-2.37	5.62	0.39	-0.46	2.19	1.54	0.96	1.69
	β	*N* (0, 1)		**0.0065**	**< 0.0001**		0.3452	0.8519		0.9090	0.9120
		Z^P		**< 0.0001**	**< 0.0001**		0.3809	0.3282		0.8169	0.8870
t = 20000	Mean		0.87	-12.34	13.60	0.66	-3.43	4.97	0.10	-1.14	1.37
	Standard deviation		0.73	0.77	0.83	0.74	0.93	0.95	0.87	0.82	0.76
	Percentile	5%	-0.31	-13.54	12.38	-0.50	-5.02	3.47	-1.33	-2.41	0.29
		95%	2.14	-11.11	14.82	1.86	-1.87	6.43	1.40	0.19	2.60
	β	*N* (0, 1)		**< 0.0001**	**< 0.0001**		**0.0367**	**< 0.0001**		0.7055	0.6265
		Z^P		**< 0.0001**	**< 0.0001**		**0.0003**	**0.0001**		0.5668	0.5115
