# Supplementary material for: Human microbiome privacy risks associated with summary statistics
Source: PLoS One. 2021 Apr 2;16(4):e0249528. doi: 10.1371/journal.pone.0249528 (PMC8018636; doi:10.1371/journal.pone.0249528)
Supplement: S4 Table — Type II error probabilities less than 0.05 are in bold. (PDF) [file pone.0249528.s011.pdf]

S4 Table. Summary statistics of simulation results obtained under the assumption that the population OTU frequencies follow a $\boldsymbol{Beta(1,1)}$ distribution. Type II error probabilities less than 0.05 are in bold.

			$n_R = n_C = 10$			$n_R = n_C = 100$			$n_R = n_C = 1000$		
			Z^P	Z^{R+}	Z^{C+}	Z^P	Z^{R+}	Z^{C+}	Z^P	Z^{R+}	Z^{C+}
t = 20	Mean		-0.19	-1.07	-0.12	-1.20	-1.29	-1.17	0.58	0.25	0.58
	Standard deviation		1.06	0.68	1.01	0.84	0.79	0.89	1.06	0.95	0.93
	Percentile	5%	-1.61	-1.61	-1.61	-2.60	-2.60	-2.60	-1.05	-1.05	-0.84
		95%	1.61	0.38	1.61	0.00	0.00	0.18	2.26	2.26	2.26
	β	*N* (0, 1)		0.7979	0.9368		0.6591	0.9998		0.9678	0.8523
		Z^P		0.7696	0.9309		0.9352	0.8783		0.8964	0.9317
t = 200	Mean		-0.52	-1.67	1.02	-0.42	-0.80	0.16	0.32	0.01	0.30
	Standard deviation		0.83	0.77	0.83	0.96	0.89	0.98	0.86	0.81	0.81
	Percentile	5%	-1.83	-2.79	-0.31	-1.85	-2.12	-1.47	-1.15	-1.27	-0.93
		95%	0.91	-0.15	2.46	1.12	0.70	1.71	1.63	1.49	1.76
	β	*N* (0, 1)		0.4495	0.7716		0.8035	0.9249		0.9690	0.9313
		Z^P		0.5327	0.4479		0.8602	0.8246		0.9014	0.9290
t = 2000	Mean		-0.53	-4.87	3.83	-0.02	-1.26	1.34	0.08	-0.29	0.57
	Standard deviation		0.91	0.71	0.86	0.81	0.73	0.78	0.83	0.80	0.80
	Percentile	5%	-1.93	-6.15	2.56	-1.33	-2.28	-0.02	-1.52	-1.57	-0.75
		95%	0.96	-3.85	5.21	1.35	-0.03	2.64	1.27	1.17	1.86
	β	*N* (0, 1)		**< 0.0001**	**0.0063**		0.6803	0.6513		0.9458	0.8893
		Z^P		**< 0.0001**	**0.0001**		0.5215	0.5112		0.9238	0.7831
t = 20000	Mean		1.59	-11.63	14.26	-0.01	-3.89	4.32	-0.49	-1.61	0.94
	Standard deviation		0.74	0.75	0.80	0.76	0.78	0.83	0.75	0.79	0.85
	Percentile	5%	0.48	-12.85	13.07	-1.04	-5.31	3.08	-1.79	-3.10	-0.46
		95%	2.68	-10.41	15.32	1.13	-2.70	5.46	0.77	-0.27	2.36
	β	*N* (0, 1)		**< 0.0001**	**< 0.0001**		**0.0033**	**0.0004**		0.5355	0.7710
		Z^P		**< 0.0001**	**< 0.0001**		**0.0001**	**< 0.0001**		0.6139	0.4200
